# Same storm, different boats: can the UK recapture improving life expectancy trends?

**DOI:** 10.1177/01410768211033895

**Published:** 2021-07-20

**Authors:** Lucinda Hiam, Parth Patel

**Affiliations:** 1 School of Geography and the Environment, University of Oxford, Oxford OX1 2JD, UK; 2 Centre for Public Health Data Science, University College London, London, WC1E7HT, UK

The COVID-19 pandemic has led to devastating loss of life and livelihood across the world. It has resulted in falls in life expectancy, a widely used summary measure of contemporary age-specific mortality rates, in most European countries.^1^ In England and Wales, life expectancy has fallen by 0.9 and 1.2 years for women and men, respectively, when compared to 2019.^2^ These falls are, however, part of a longer-term trend that began long before the COVID-19 pandemic. For the past decade, life expectancy in the UK has been stalling, and falling in some regions and social groups, and inequality in age at death has been rising.^3^ In this piece, we consider the health of the UK population before the pandemic, compare this to the health of the populations in the USA and Japan, as investigated in a recent paper,^3^ and ask if it is possible to recapture previous improving life expectancy trajectories.

## Health in Britain before 2020

The pandemic arrived in the UK after a decade of austerity and declining health outcomes: stalling life expectancy, rising infant mortality rates and widening health inequalities.^4,5^ Spending on health and social care did not keep pace with rising demand, leading to declining service access and outcomes.^4,6^ A phenomenon first described in the USA of rising mid-age mortality (aged 45–54 years) from ‘deaths of despair’ – that is deaths from suicide, drug and alcohol overdoses, and alcoholic liver disease – has now been observed in England and Wales^7^ and in Scotland,^8,9^ with growing evidence that an increase in mortality at mid-age is contributing to a widening of inequalities in the age of death in the UK.^3,10^

This alarming loss of life did not occur suddenly. In 2015, when the UK saw one of the largest increases in year-on-year deaths since World War II, alarm bells were sounded but the government response was not one of national crisis.^11^ Health outcomes continued deteriorating in the years that followed, and many began to suggest the declines could be a result of austerity policies that were resulting in the slowing down or cutting of government expenditure on health, social care, education, welfare and housing. The Cameron government, which believed austerity was good economic policy, refuted this link and ignored widespread concern from public health experts.^12^

While other causes may also have played a role in the poor state of population health in the UK prior to the pandemic, such as reductions in improving cardiovascular disease mortality (which may itself be related to austerity),^13^ the evidence on the contribution of austerity policies on health outcomes is overwhelming.^14^ As a result, the UK ‘limped into the pandemic … an unhealthy population marked by growing inequalities and a worsening of the conditions in which people are born, grow, live, work and age’.^15^

## Same storm, different boats

Japan and the USA – two countries with very different experiences of the COVID-19 pandemic – entered the pandemic with very different population health patterns. The USA had seen unprecedented falls in life expectancy every year since 2015, preceded by rising lifespan variation,^16^ representing increasing inequality in the age at death. Japan, in contrast, had sustained increases in life expectancy and reductions in lifespan variation, with no worsening of health inequalities, despite a decade of low economic growth.^3,17^

Several factors, including government policy during the pandemic, determine the severity of COVID-19 in any given country. There appears to be, however, an observable difference between the high-income countries examined that entered the pandemic with deteriorating population health and growing inequalities, such as the UK and USA, and high-income countries where health is more equally distributed across the population, such as Japan.^18^

Could it be that countries that entered the pandemic on a ‘healthy trajectory’, with stable or improving health outcomes across the population, were more resilient to the pandemic?

## The road not taken

In the past year, life expectancy in England and Wales has fallen,^2^ superimposed on a worsening trend before the pandemic.^3,4^ Without urgent intervention, the UK is unlikely to recapture the trajectory of health in countries like Japan.

For decades, public health practitioners and academics have been calling for greater action on the wider determinants of health, such as education, employment and housing.^19^ The recently established Office for Health Promotion,^20^ with a much overdue cross-government ministerial board, aims to operationalise a ‘step change in public health policy’. It remains to be seen if this is substantial or symbolic, but it is clear the Office will fail to meet the Conversative Party pledge to increase healthy life expectancy by five years by 2035,^21^ if it does not explicitly consider the role of socioeconomic deprivation in shaping health.

In order to recapture improving life expectancy trends, public services – including health and social care services – need investment and reform, not least to handle the COVID-19 aftershocks. The Institute for Public Policy Research has called for an average £12 billion per annum uplift to NHS,^22^ social care and public health services expenditure, while the LSE-*Lancet* commission has called for an average £10.2 billion per annum for the next decade.^6,22^ Instead, analysis of November 2020 and March 2021 Budgets by the Institute for Fiscal Studies reveals austerity policies by stealth in several government departments, most notably in funding for local government which is facing a 3% real-terms cut in 2022–2023.^23^ The Institute for Fiscal Studies concluded ‘if [the proposed figures] are adhered to then many public services are due a second, sharp dose of austerity’.^24^

Japan and the USA are taking different approaches. Both have adopted more Keynesian responses to the economic crisis brought about by the pandemic and have significantly increased government expenditure. This is in keeping with a shift in macroeconomic consensus, with even eminent institutions of fiscal discipline such as the International Monetary Fund and the Organisation for Economic Co-operation and Development, advocating a sizeable debt-financed fiscal stimulus.^25,26^ The UK’s spending plans in response to the COVID-19 pandemic are less than half of the USA’s and one-third of Japan’s, adjusted to the size of the economy.^27^ President Biden has since committed to a further $4tn spending over the next decade, which includes significant investment in health and social care, child care, education and housing.^28,29^ It will be of great interest to observe the impact this has on the current declining life expectancy trends in the USA.

## Conclusion

As we wait for an official COVID-19 inquiry, the question remains: how prepared was the UK for a pandemic? The health of the population was deteriorating before 2020, with a loss of previous improving trends in life expectancy. It is not certain what role this played, but what is abundantly clear is the impact of the poor and unequal distribution of health in the UK prior to the pandemic: COVID-19 has not just fed on these inequalities, it was amplified by them.

The road to recovery for health, including life expectancy trends, requires an ideological change in social and economic policy making. It requires co-ordinated and collaborative action across local and national government, and greater investment in public services and welfare. Health is a public good – it must now become a marker of societal progress.

## Declarations

**Ethics approval:** Not applicable.

**Guarantor:** LH

**Contributorship:** LH conceived the first draft which PP significantly edited and added to.

## References

[1] Eurostat. *Life expectancy decreased in 2020 across the EU 2021.* See https://ec.europa.eu/eurostat/web/products-eurostat-news/-/edn-20210407-1 (last checked 8 April 2021).

[2] Aburto JM, Kashyap R, Schöley J, Angus C, Ermisch J, Mills MC, Dowd JB. Estimating the burden of the COVID-19 pandemic on mortality, life expectancy and lifespan inequality in England and Wales: a population-level analysis. *J Epidemiol Community Health* 2021 Jan 19:jech-2020-215505. doi: 10.1136/jech-2020-215505. Epub ahead of print. PMID: 33468602; PMCID: PMC7818788. 10.1136/jech-2020-215505 PMC7818788 33468602

[3] Hiam L, Minton J, McKee M. What can lifespan variation reveal that life expectancy hides? Comparison of five high-income countries. *J R Soc Med* 2021 May 6:1410768211011742. doi: 10.1177/01410768211011742. Epub ahead of print. PMID: 33955790. 10.1177/01410768211011742 PMC8358556 33955790

[4] Hiam L Dorling D McKee M . Things fall apart: the British Health Crisis 2010-2020. Br Med Bull 2020; 133: 4–15. 32219417 10.1093/bmb/ldz041

[5] Robinson T Brown H Norman PD Fraser L Barr B Bambra C . The impact of New Labour’s English health inequalities strategy on geographical inequalities in infant mortality: a time-trend analysis. J Epidemiol Commun Health 2019; 73: 564–568. 10.1136/jech-2018-211679 30890592

[6] Anderson M, Pitchforth E, Asaria M, Brayne C, Casadei B, Charlesworth A et al. LSE-Lancet Commission on the future of the NHS: re-laying the foundations for an equitable and efficient health and care service after COVID-19. *Lancet*. 2021 May 22;397(10288):1915-1978. doi: 10.1016/S0140-6736(21)00232-4. Epub 2021 May 6. 10.1016/S0140-6736(21)00232-4 33965070

[7] Joyce R Xu X . Inequalities in the Twenty-first Century: Introducing the IFS Deaton Review, London: The Institute for Fiscal Studies, 2019.

[8] Allik M Brown D Dundas R Leyland A . Deaths of despair: cause-specific mortality and socioeconomic inequalities in cause-specific mortality among young men in Scotland. Int J Equity in Health 2020; 19: 215–215. 33276793 10.1186/s12939-020-01329-7 PMC7716282

[9] Walsh D, McCartney G, Minton J, Parkinson J, Shipton D, Whyte B. Deaths from 'diseases of despair' in Britain: comparing suicide, alcohol-related and drug-related mortality for birth cohorts in Scotland, England and Wales, and selected cities. *J Epidemiol Community Health*. 2021 May 27:jech-2020-216220. doi: 10.1136/jech-2020-216220. Epub ahead of print. PMID: 34045325. 10.1136/jech-2020-216220 PMC8588300 34045325

[10] Darlington-Pollock F Norman P . Stalling life expectancy and increased mortality in working ages deserve urgent attention. Lancet Public Health 2019; 4: e543–e544. 31677771 10.1016/S2468-2667(19)30207-5

[11] Dorling D. How many more will be dead by Christmas? *London Review of Books* 5 October 2020 https://www.lrb.co.uk/blog/2020/october/how-many-more-will-be-dead-by-christmas accessed 8 July 2021.

[12] Hiam L Dorling D McKee M . Rise in mortality – when will the government take note? BMJ 2018; 361: k2747–k2747. 29941609 10.1136/bmj.k2747

[13] Murphy M, Luy M and Torrisi O. *Stalling of mortality in the United Kingdom and Europe: an analytical review of the evidence London School of Economics.* See www.lse.ac.uk/social-policy/Assets/Documents/PDF/working-paper-series/11-19-Mike-Murphy.pdf (2019, last checked 5 December 2020).

[14] Darlington-Pollock F, Green MA, Simpson L. Why were there 231 707 more deaths than expected in England between 2010 and 2018? An ecological analysis of mortality records. *J Public Health (Oxf)*. 2021 Mar 26:fdab023. doi: 10.1093/pubmed/fdab023. Epub ahead of print. PMID: 33765120; PMCID: PMC8083632. 10.1093/pubmed/fdab023 PMC8083632 33765120

[15] Marmot M. *Why did England have Europe's worst Covid figures? The answer starts with austerity 2020.* See www.theguardian.com/commentisfree/2020/aug/10/england-worst-covid-figures-austerity-inequality (last checked 22 September 2020).

[16] van Raalte AA Sasson I Martikainen P . The case for monitoring life-span inequality. Science 2018; 362: 1002–1004. 30498117 10.1126/science.aau5811

[17] Hiyoshi A Honjo K Platts L Suzuki Y . OP86 Low economic growth, health, health inequalities and sustainable development goals in a rich country: 27-year Japanese time series. J Epidemiol Commun Health 2020; 74(Suppl 1): A40–A41.

[18] Hasell J. *Which countries have protected both health and the economy in the pandemic? Our World in Data2020.* See https://ourworldindata.org/covid-health-economy (last checked 22 September 2020).

[19] Marmot M, Allen J, Goldblatt P, Herd E, Morrison J (2020). Build Back Fairer: The COVID-19 Marmot Review. The Pandemic, Socioeconomic and Health Inequalities in England. London: Institute of Health Equity.

[20] Department of Health and Social Care. *New Office for Health Promotion to Drive Improvement of Nation’s Health 2021.* See www.gov.uk/government/news/new-office-for-health-promotion-to-drive-improvement-of-nations-health (last checked 10 May 2021).

[21] The Conservative and Unionist Party. *Get Brexit Done. Unleash Britain’s Potential 2019*. See https://assets-global.website-files.com/5da42e2cae7ebd3f8bde353c/5dda924905da587992a064ba_Conservative%202019%20Manifesto.pdf (last checked 5 May 2021).

[22] Patel P, Thomas C, and Quilter-Pinner H. *State of Health and Care Institue of Public Policy Research 2021.* See www.ippr.org/news-and-media/press-releases/12-billion-booster-shot-needed-to-make-nhs-and-social-care-fit-for-future-after-pandemic-landmark-report (last checked 8 April 2021).

[23] Zaranko B. *The Chancellor’s Spending Plans are Even Tighter than They Seem: Institute for Fiscal Studies*. See www.ifs.org.uk/publications/15365 (last checked 5 May 2021).

[24] Tolhurst A. *Public Services Face “Second Dose Of Austerity” To Meet Rishi Sunak’s Spending Cuts, Warns IFS 2021.* See www.politicshome.com/news/article/ifs-budget-2021-rishi-sunak-public-services-new-wave-austerity (last checked 5 May 2021).

[25] International Monetary Fund. *Fiscal Monitor: Policies for the Recovery 2020.* See www.imf.org/en/Publications/FM/Issues/2020/09/30/october-2020-fiscal-monitor (last checked 10 May 2021).

[26] Giles C. OECD warns governments to rethink constraints on public spending. *Financial Times* 2021. See www.ft.com/content/7c721361-37a4-4a44-9117-6043afee0f6b (last checked 10 May 2021).

[27] Jung C, Dibb G and Patel P. *Boost it like Biden: Why the UK needs an Economic Stimulus Four Times the Size Currently Planned Institute for Public Policy Research 2021.* See www.ippr.org/research/publications/boost-it-like-biden (last checked 10 May 2021).

[28] The White House. *FACT SHEET: The American Jobs Plan 2021.* See www.whitehouse.gov/briefing-room/statements-releases/2021/03/31/fact-sheet-the-american-jobs-plan/ (last checked 10 May 2021).

[29] The White House. *FACT SHEET: The American Families Plan 2021*. See www.whitehouse.gov/briefing-room/statements-releases/2021/04/28/fact-sheet-the-american-families-plan/ (last checked 10 May 2021).

